# Rotavirus outbreaks in China, 1982–2021: a systematic review

**DOI:** 10.3389/fpubh.2024.1423573

**Published:** 2024-08-08

**Authors:** Yi Tian, Fan Yu, Guanhua Zhang, Chunyu Tian, Xinxin Wang, Yanwei Chen, Hanqiu Yan, Lei Jia, Daitao Zhang, Quanyi Wang, Zhiyong Gao

**Affiliations:** ^1^Institute for Infectious Disease and Endemic Disease Control, Beijing Center for Disease Prevention and Control, Beijing, China; ^2^School of Public Health, The University of Hong Kong, Pokfulam, Hong Kong SAR, China; ^3^Liver Research Center, Beijing Friendship Hospital, Capital Medical University, Beijing, China; ^4^Department of Allergy, Children’s Hospital Affiliated with the Capital Institute of Pediatrics, Beijing, China; ^5^School of Public Health, Capital Medical University, Beijing, China

**Keywords:** rotavirus, outbreak, epidemiological characteristics, transmission, genotypes

## Abstract

**Background:**

Rotavirus is globally recognized as an important cause of acute gastroenteritis in young children. Whereas previous studies focused more on sporadic diarrhea, the epidemiological characteristics of rotavirus outbreaks have not been systematically understood.

**Methods:**

This systematic review was carried out according to the Preferred Reporting Items for Systematic Review and Meta-Analysis standards, WANFANG, China National Knowledge Infrastructure (CNKI), PubMed, and Web of Science databases were searched from database inception to February 20, 2022. We used SPSS 21.0 statistical software for data analysis, RStudio1.4.1717, and ArcGIS trial version for plotting bar graphs and maps.

**Results:**

Among 1,596 articles, 78 were included, with 92 rotavirus outbreaks and 96,128 cases. Most outbreaks (67.39%, 62/92) occurred in winter and spring. The number of rotavirus outbreaks reported in the eastern region was more than that in the western region. Outbreaks were most commonly reported in villages (33/92, 35.87%), followed by hospitals (19, 20.65%). The outbreak duration was longer in factories and workers’ living places, and villages, while it was shorter in hospitals. Waterborne transmission was the main transmission mode, with the longest duration and the largest number of cases. Rotavirus groups were identified in 66 outbreaks, with 40 outbreaks (60.61%) caused by Group B rotaviruses and 26 outbreaks (39.39%) caused by Group A rotaviruses. Significant differences were found in duration, number of cases, settings, population distribution, and transmission modes between Groups A and B rotavirus outbreaks.

**Conclusion:**

Rotavirus is an important cause of acute gastroenteritis outbreaks in China. It should also be considered in the investigation of acute gastroenteritis outbreaks, especially norovirus-negative outbreaks.

## 1 Background

Acute gastroenteritis accounts for a significant portion of global morbidity and mortality. Norovirus is the most common pathogen of acute gastroenterological outbreaks, and outbreaks caused by rotavirus are also common (1). Rotavirus is a non-enveloped virus classified within the *Reoviridae* family. The 11 segments of double-stranded RNA residing in the core encode six viral proteins (VP1-VP4, VP6, and VP7) that make up the viral capsid, along with six non-structural proteins (NSP 1–6). According to the different VP6 antigenicity, rotavirus was divided into 10 groups (A-J). Groups A, B, C, and H infect humans and animals, while other groups only infect animals (2). Group A rotavirus is prevalent worldwide, especially among children (3). Group B rotavirus appears to have been reported only in Asia in the 1980s (4). At the same time, enteric infections caused by Group C and H rotaviruses have been rarely reported.

Previous rotavirus surveillance and studies have focused on sporadic diarrhea, especially in children under 5 years old. Many studies have reported the epidemiological characteristics, disease burden, clinical manifestation, and genotype distribution of rotavirus diarrhea among children in China and other countries (5–7). In China, sentinel surveillance of rotavirus in hospitalized children under 5 years of age with diarrhea began in 1998. In 2009, China joined the Global Rotavirus Surveillance Network. By 2021, China’s rotavirus sentinel surveillance network has covered 42 sentinel hospitals in 31 provinces (autonomous regions and municipalities), and the surveillance objects have been extended to children and adults with diarrhea in outpatient and inpatient settings (8). A meta-analysis showed that the overall rate of rotavirus diarrhea in children under 5 years of age was 34.0% (95%CI: 31.3–36.8%) during 2011–2018, with differences in rotavirus detection rates among different regions in China (9). Outbreaks caused by rotavirus are also a component of measuring the overall disease burden of rotavirus. However, the epidemiological characteristics of rotavirus outbreaks have not been systematically analyzed in China. Therefore, this study collected data about rotavirus outbreaks reported in the literature from 1982 to 2021 to characterize rotavirus outbreaks in terms of temporal distribution, regional distribution, population distribution, settings, transmission modes, clinical symptoms of cases, and genotypes in China.

## 2 Methods

### 2.1 Search strategy

This systematic review was conducted following the Preferred Reporting Items for Systematic Reviews and Meta-Analyses (PRISMA) checklist. We identified “rotavirus” and “outbreak” or “exposure” and “China” as the keywords, and searched for articles on PubMed, Web of Science databases, China National Knowledge Infrastructure (CNKI), and Chinese Wan Fang digital database (WANFANG) from the inception of the databases to February 20, 2022.

### 2.2 Selection and exclusion criteria

Studies had to meet all four of the following criteria for inclusion: (1) were published in Chinese or English; (2) three or more cases with an epidemiological association can be defined as an outbreak; (3) provided at least three of the following information: outbreak time, region, the number of cases, number of persons exposed, attack rates, settings, transmission modes, genotypes; (4) rotavirus antigen or nucleic acid could be detected in at least two patients’ stool specimens, or rotavirus IgM antibodies could be detected in at least two patients’ serum specimens. As in the co-infection outbreaks, if more rotavirus samples were detected in specimens than other pathogens, we supposed that rotavirus infection should be the main diarrheal pathogen in these outbreaks.

Studies should be excluded if they meet one of the following criteria: (1) were not available for full-text or valid data could not be collected from the abstract; (2) the subjects were not humans; (3) suspected rotavirus outbreaks without enough laboratory evidence; (4) were review or meta-analysis articles.

### 2.3 Data processing and analysis

Repeated outbreaks were counted only once, and the same information that appeared in different studies was summarized and integrated. If one article contained two or more outbreaks, each outbreak should be counted separately. The following information was extracted from each study: article information (title, geographical position, author, journal name, time of publication), outbreak terms (time, region, number of cases, number of persons exposed, attack rates, settings, transmission modes, genotypes). The definition of hospital outbreaks is that the index cases and all other cases not experience diarrhea before admission, and the outbreaks occurred in the hospital. Studies searching, selection and exclusion, and data extraction were carried out independently by two individuals, and inconsistent results were then arbitrated by the third person.

### 2.4 Statistical analysis

Endnote X9 was used to import and manage retrieved records. WPS Office was used to establish an epidemiological information database of rotavirus outbreaks. RStudio1.4.1717^1^ and the ArcGIS trial version were used for plotting bar graphs and maps. A map of China is available at the Resource and Environment Science and Data Center.^2^ SPSS 21.0 statistical software was used to conduct data analysis. A chi-square test was performed to compare the rates between different groups. The Wilcoxon rank-sum test was used to analyze differences in the number of rotavirus outbreaks between different regions, seasons, and genotypes. The Kruskal-Wallis H test was calculated to compare the differences in epidemiological characteristics between rotavirus outbreaks in different settings or by different transmission modes. Statistical significance was assumed for *p* < 0.05 (two-tailed).

## 3 Results

### 3.1 Literature screening and basic information

**Figure 1:**
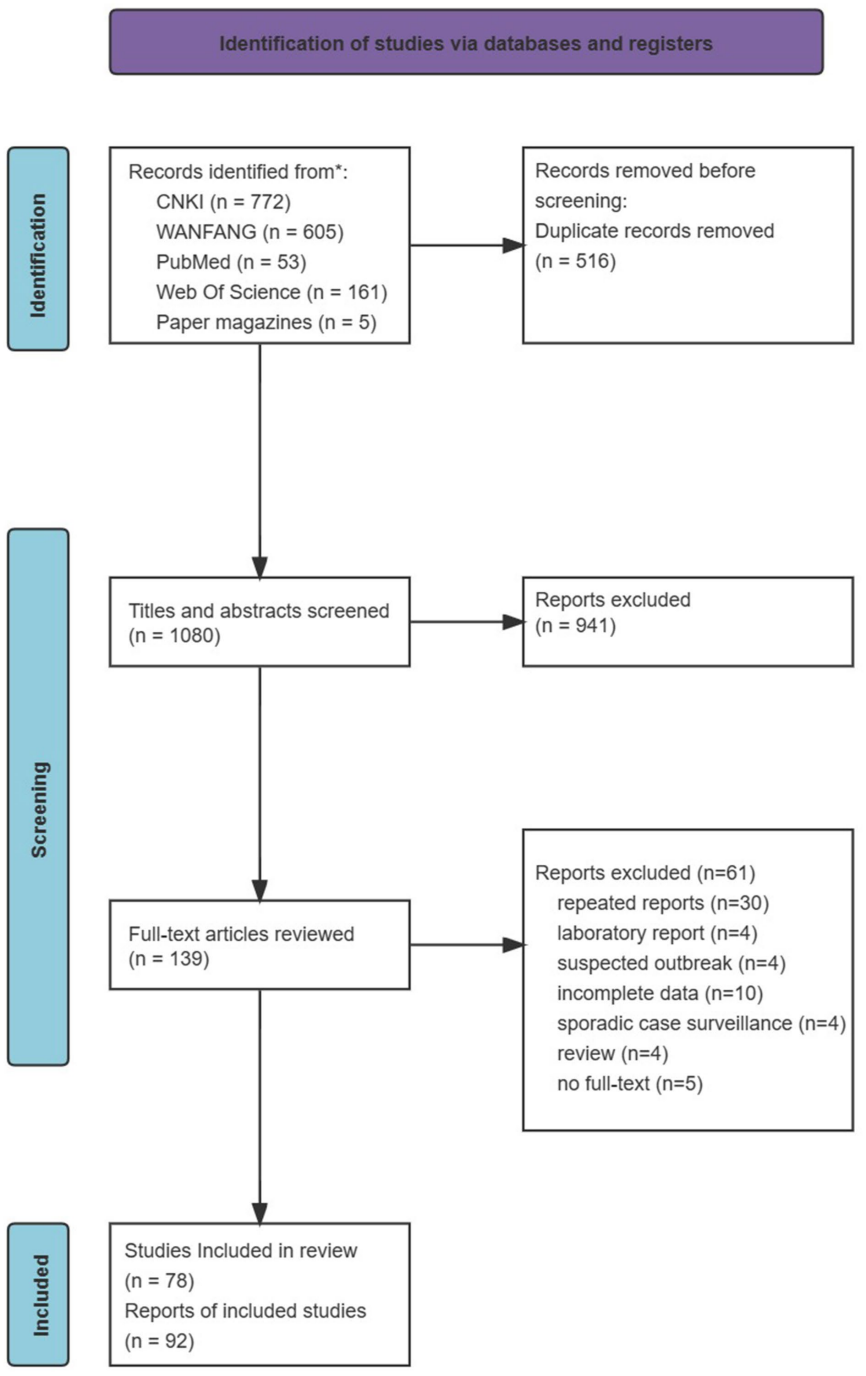
PRISMA flowcharts showing the selection process.

In total, 2,314 articles were initially identified. Of them, 1,080 were unique records, and 941 were excluded based on a title and abstract review (see Figure 1). A total of 139 full-text articles were assessed for eligibility. From these, 30 were duplicate reports, 4 reported laboratory data only (with no epidemiological data), 4 were not identified as rotavirus outbreaks, 10 had incomplete data, 4 belonged to sporadic case surveillance, 4 were reviews, and 5 did not have full text. Finally, 78 articles were included.

A total of 92 rotavirus outbreaks were reported in the 78 included articles. The outbreaks occurred between November 1982 and March 2021. Ninety-two rotavirus outbreaks involved 96,128 cases, with a median of 140 cases per outbreak (ranging from 3 to 20,000). The duration was reported for 72 outbreaks, with a median of 18 days (ranging from 2 to 154 days). The attack rates were reported for 64 outbreaks and ranged from 0.65% to 85.34%, with a median of 17.55%. The outbreak with the fewest cases occurred in a hospital in Jiangxi province in April 2013, involving 3 cases, and lasting 3 days. The largest reported outbreak occurred in a county in Anhui province in 1983, affecting 20,000 cases, and lasting for 4 months. It was speculated that the water source was polluted due to heavy rainfall.

### 3.2 Temporal distribution

**Figure 2:**
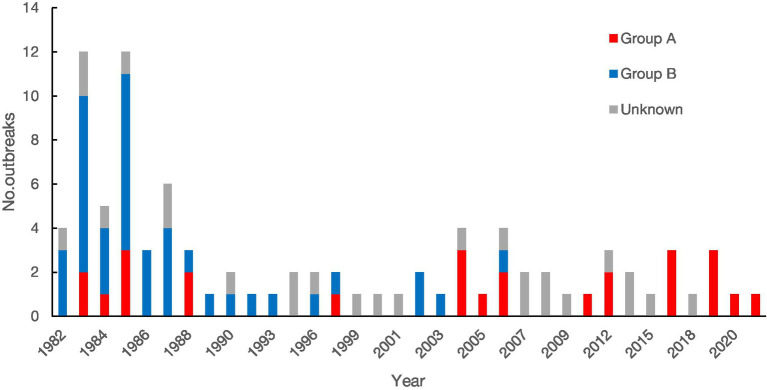
Yearly and group distribution of rotavirus outbreaks reported in China, 1982–2021.

A total of 91 rotavirus outbreaks were documented with year and month information. One outbreak was reported in the spring of 1983 but did not provide monthly information. The earliest outbreak occurred in November 1982, and the latest occurred in March 2021. Forty-six outbreaks (50.00%) were reported during 1982 to 1989 (see Figure 2). The number of outbreaks peaked in 1983 with 13 outbreaks. The average interval between the occurrence and publication of the 92 outbreaks was 2 years.

**Figure 3:**
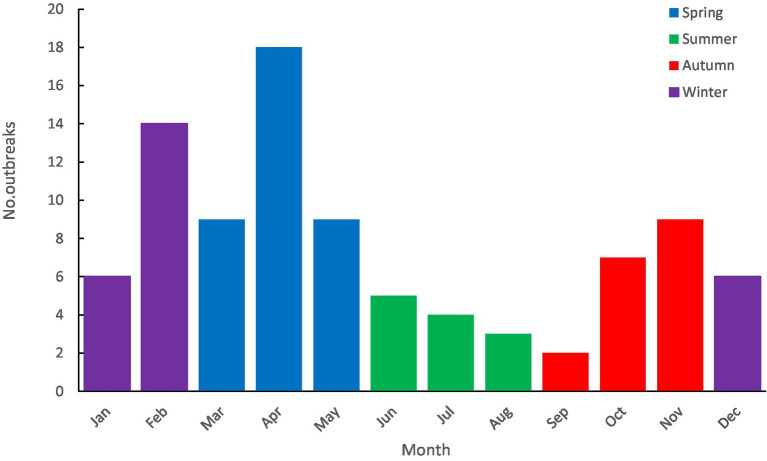
Monthly distribution of rotavirus outbreaks reported in China, 1982–2021.

Rotavirus outbreaks occurred every month, with the highest number of outbreaks in April (18 outbreaks) and the lowest in September (2 outbreaks) (see Figure 3). There were 36 outbreaks in spring (March to May), 12 in summer (June to August), 18 in autumn (September to November), and 26 in winter (December to February). Most outbreaks occurred in winter and spring, accounting for 67.39% (62/92). The median duration of outbreaks (interquartile range, IQR) in the epidemic season (winter and spring) and the non-peak season (summer and autumn) were 18 (11, 32) days and 19 (7, 51) days, respectively. Statistical analysis showed no statistical significance (*W* = 1,696.500, *p* = 0.822) between the durations.

### 3.3 Regional distribution

**Figure 4:**
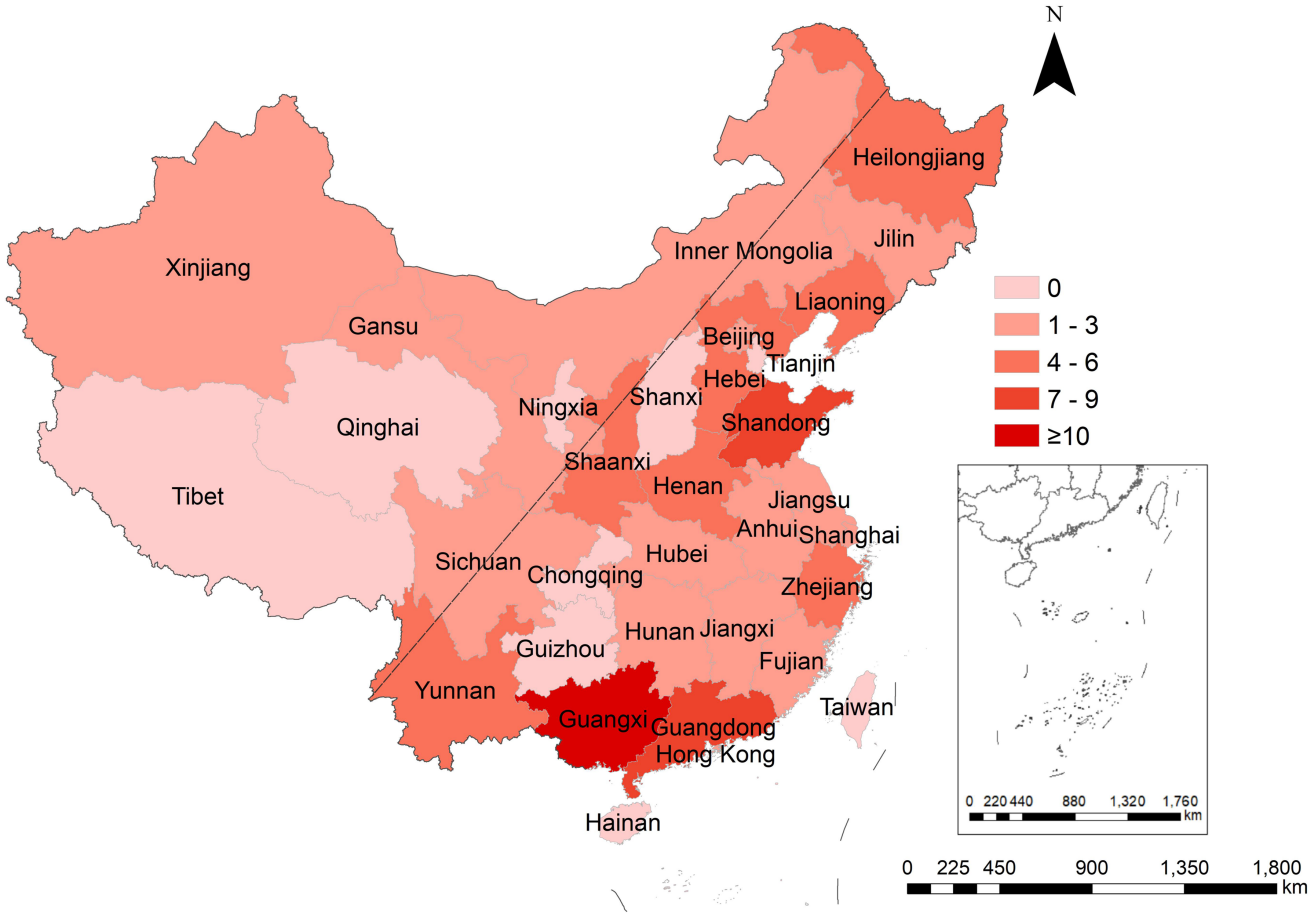
Regional distribution of rotavirus outbreaks reported in China, 1982–2021. The “Hu line” was used to divide China into eastern and western regions.

Ninety-two outbreaks stated the occurrence areas, including 18 provinces, 2 municipalities (Beijing and Shanghai), 3 autonomous regions, and Hong Kong Special Administrative Region (see Figure 4). Guangxi Zhuang autonomous region reported the most outbreaks (*n* = 15), followed by Guangdong (*n* = 8) and Shandong (*n* = 7). Referring to Wu Ruijun and Zhu Baoshu’s method (10), we used the “Hu line” to divide China into eastern and western regions and found that more outbreaks were reported in the east (*n* = 88) than in the west (*n* = 4) (*W* = 4,092.00, *p* < 0.001).

### 3.4 Population distribution and clinical symptoms

Information on the population distribution was available for 89 outbreaks. Among these outbreaks, 42 occurred in the general population, 28 involved only children, and 19 involved only adults. The numbers of male and female cases were reported in 42 outbreaks, with 7,520 male cases and 6,389 female cases, and the ratio of male to female was 1.18:1. The minimum ages of patients were available for 42 outbreaks, with a mean of 2 years, ranging from 30 min after birth to 20 years of age. The maximum ages were reported for 44 outbreaks, with a mean of 49 years, ranging from 9 days to 94 years of age. Age groups in the literature are inconsistent, and outbreaks mainly affected adults overall.

**Figure 5:**
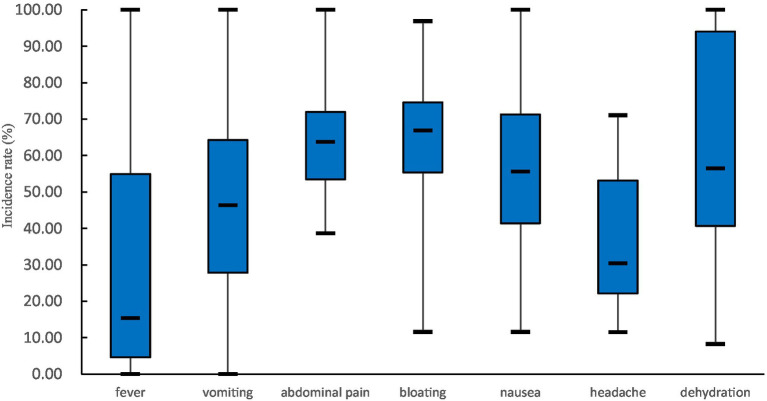
Common clinical symptoms (excluding diarrhea) of patients in rotavirus outbreaks in China, 1982–2021. The number of outbreaks involved in the analysis of each symptom is as follows: 42 in fever, 44 in vomiting, 37 in abdominal pain, 23 in bloating, 29 in nausea, 10 in headache, and 12 in dehydration.

The most common symptom of cases was diarrhea. Diarrhea incidences were reported in 65 outbreaks, with 100% in 56 outbreaks. In the remaining 9 outbreaks, the incidences of diarrhea ranged from 45 to 85% in 6 outbreaks and was more than 90% in 3 outbreaks. Other common clinical symptoms included abdominal pain, bloating, nausea, headache, and vomiting (see Figure 5). Less common symptoms such as dizziness, fatigue, chills, and belching were also reported. The incidences of dehydration were reported in 12 outbreaks, with the median [interquartile range] being 56.45% (40.70%, 94.02%). A total of 22 deaths were reported in 5 outbreaks, with 4 cases dying from severe dehydration in 2 outbreaks. The cause of death for the remaining 18 deaths was not reported. Sixteen death cases were reported in an outbreak reported in Fujian province in 1982, and the case fatality rate was 6.13% (16/261). After 2004, there have been no reported deaths in rotavirus outbreaks.

### 3.5 Settings and transmission modes

The majority of outbreaks occurred in villages (33/92, 35.87%). Outbreaks were also reported in hospitals (19, 20.65%), factories and workers’ living places (14, 15.22%), schools (10, 10.87%), armies (6, 6.52%), communities (3, 3.26%), welfare home (1, 1.09%) and restaurants (1, 1.09%). Five outbreaks did not provide setting information.

Significant differences were found between outbreaks in different settings in terms of duration (*H* = 26.997, *p* < 0.001), and number of cases (*H* = 35.726, *p* < 0.001). No statistically significant differences were identified in outbreak attack rates (*H* = 12.313, *p* = 0.055). Outbreaks that occurred in factories and workers’ living places and villages had a longer duration, while outbreaks in hospitals and armies had a shorter duration. The number of cases in factories and workers’ living places was the greatest (see Table 1).

**Table 1:** Characteristics between outbreaks of different settings and transmission modes.

Groups	Outbreak duration, *N*, Median [IQR] (d)^a^	Cases, *N*, Median [IQR] (*n*)^a^	Attack rate, *N*, Median [IQR] (%)^a^
Settings			
Villages	26, 27.00 [17.50, 54.00]	33, 163.00 [63.00, 641.00]	24, 17.34 [9.70, 33.91]
Hospitals	19, 7.00 [6.00, 12.00]	19, 11.00 [6.00, 29.00]	11, 26.71 [17.78, 43.40]
Factories and workers’ living place	10, 33.50[18.75, 53.50]	14, 468.00 [149.00, 4937.00]	12, 14.28 [11.47, 18.72]
Schools	8, 15.50 [9.00, 19.00]	10, 219.00 [51.00, 453.00]	8, 5.67 [2.11, 16.42]
Armies	6, 11.50 [8.25, 21.00]	6, 220.00 [22.00, 725.00]	6, 34.30 [9.52, 46.99]
Others settings	4, 14.50 [4.50, 46.25]	5, 133.00 [12.00, 345.00]	4, 41.95 [12.14, 53.46]
Unknown	4, 53.50 [22.00, 130.75]	5, 1000.00 [270.00, 11006.00]	2, 15.87 [13.00, −]
Transmission modes			
Waterborne transmission	36, 34.00 [18.00, 51.00]	48, 340.00 [149.00, 1211.00]	34, 14.97 [10.97, 33.02]
Person-to-person transmission	26, 10.00 [6.00, 17.50]	27, 18.00 [8.00, 69.00]	17, 26.03 [9.88, 35.08]
Others transmissions	8, 18.00 [7.50, 58.00]	8, 77.00 [19.00, 152.00]	6, 5.60 [1.19, 25.78]
Unknown	7, 16.00 [7.00, 61.00]	9, 98.00 [33.00, 3785.00]	5, 33.91 [2.18, 45.26]

a IQR, Inter Quartile Range.

The modes of transmission were reported for 83 outbreaks. The common transmission modes were waterborne (48, 57.83%) and person-to-person (27, 32.53%). Three outbreaks (3.61%) involved foodborne transmission, three (3.61%) involved multiple transmission modes, and two (2.41%) did not exclude the possibility of respiratory transmission. In three foodborne outbreaks, one outbreak was caused by a co-infection of pathogenic *Escherichia coli* and rotavirus, and the risk factor analysis indicated that the suspected food was crayfish. The second outbreak occurred in a school, the school canteen’s environmental hygiene was poor, and the plates were not cleaned in time. Combined with the clinical presentation of the case and laboratory findings, it was inferred that the outbreak was caused by an unclean diet. In another outbreak, the first case was a cafeteria worker who continued to work after becoming ill, causing diarrhea in students who had meals. Although rotavirus was not detected in the sampled food, it was presumed to be foodborne.

Waterborne transmission primarily occurred in villages (21), factories and workers’ living places (13). Person-to-person transmission mainly occurred in hospitals (16), villages (4), and schools (3).

There are significant differences in outbreak duration (*H* = 21.025, *p* < 0.001) and the number of cases (*H* = 34.948, *p* < 0.001) among the different transmission modes, but there is no significant difference in attack rates (*H* = 2.461, *p =* 0.482). Waterborne outbreaks had the longest duration and highest number of cases. Person-to-person transmission outbreaks had the shortest duration and the lowest number of cases, but the highest attack rate (excluding outbreaks of unknown transmission modes).

### 3.6 Groups and genotypes

Rotavirus groups were identified in 66 outbreaks. Group A rotaviruses caused 26 outbreaks (39.39%), while Group B rotaviruses caused 40 outbreaks (60.61%). Genotypes were available in 9 Group A rotavirus outbreaks, including G1 (3), G2 (1), G1P[8] (1), G2P[4] (1), G4P[2] (1) and G9P[8] (2). Three G1 outbreaks occurred in 2004, 2005, and 2006, one G2 outbreak in 2004, one G1P[8] outbreak in 2006, two G2P[4] outbreaks in 1997 and 2017, respectively and two G9P[8] outbreaks in 2017 and 2019, respectively.

There are significant differences in duration (*W* = 357.500, *p* < 0.001) and number of cases (*W* = 407.000, *p* < 0.001) between Group A and B rotavirus outbreaks, but there were no significant differences in the attack rates (*W* = 501.000, *p =* 0.432). Most Group A rotavirus outbreaks occurred in hospitals and villages, while Group B rotavirus outbreaks mainly occurred in villages, factories, and workers’ living places. Group A rotavirus outbreaks mainly occurred in children, and Group B occurred in the general population and adults. In Group A and Group B rotavirus outbreaks, person-to-person and waterborne transmissions were most common, respectively. There are significant differences in settings (*p* < 0.001), population distribution (*χ*^2^ = 52.881, *p* < 0.001), and transmission modes (*p* < 0.001) between Group A and B rotavirus outbreaks (see Table 2).

**Table 2:** Epidemiological characteristics between Group A and B rotavirus outbreaks.

Variables	Group A	Group B	*w/χ* ^2^	*p* value
Outbreak duration, *N*, Median [IQR] (d)^a^	22, 11.50 [6.00, 19.00]	28, 34.50 [19.00, 58.50]	357.500	<0.001
Cases, *N*, Median [IQR]^a^	26, 28.00 [8.00, 106.00]	40, 610.00 [163.00, 2232.00]	407.000	<0.001
Attack rate, *N*, Median [IQR] (%)^a^	22, 18.03[4.94, 36.80]	31, 17.71 [11.25, 33.02]	501.000	0.432
**Seasons**			5.845	0.119
Spring	7	20		
Summer	8	8		
Autumn	8	5		
Winter	3	7		
**Settings**			—^b^	<0.001
Villages	6	20		
Hospitals	10	1		
Factories and workers’ living places	1	10		
Schools	5	3		
Others	4	3		
Unknown	0	3		
**Population distribution**			52.881	<0.001
Children	17	0		
Adults	2	13		
General population	4	27		
Unknown	3	0		
**Transmission modes**			—^b^	<0.001
Waterborne transmission	6	34		
Person-to-person transmission	13	4		
Other transmission	4	0		
Unknown	3	2		

a IQR, Inter Quartile Range.

b Fisher probability method results.

### 3.7 Co-infections

There were three outbreaks in this study as co-infections. One outbreak occurred in a village, the stool of 97 patients was examined, 61 specimens were positive for rotavirus, and 4 specimens were positive for both rotavirus and Toxigenic *Escherichia coli*. The second outbreak occurred in a unit in adults, 12 specimens were positive for rotavirus, and 5 specimens were positive for *Campylobacter jejuni*. Another outbreak occurred at a training base, 10 and 11 specimens were positive for pathogenic *Escherichia coli* and Group A rotavirus, respectively.

## 4 Discussion

Rotavirus is the main pathogen of sporadic acute gastroenteritis, and can also cause outbreaks of acute gastroenteritis. To get a comprehensive understanding of the rotavirus outbreaks in China, we summarized and analyzed the epidemiological characteristics of rotavirus outbreaks in China from 1982 to 2021. A total of 78 articles were included, reporting 92 rotavirus outbreaks with 96,128 cases. The median number of cases per outbreak was 140 (ranging from 3 to 20,000), and the median outbreak duration was 18 days (ranging from 2 to 154 days). The attack rates ranged from 0.65% to 85.34%, with a median of 17.55%. Waterborne transmission and person-to-person transmission were the main modes of transmission.

In this study, 46 outbreaks (50.00%) were reported between 1982 and 1989, with a peak of 13 outbreaks in 1983. This result indicates that rotavirus outbreaks are no longer a major public health issue, but they still need to be considered in the investigation of acute gastroenteritis outbreaks.

Rotavirus infections peak in winter in northern hemisphere countries and are prevalent throughout the year in subtropic countries (11, 12). In our study, rotavirus outbreaks occurred every month, with the highest incidence in winter and spring (67.39%). Rotavirus infection was negatively correlated with ambient temperature, suggesting that low temperature may be beneficial to the virus (13, 14).

The number of rotavirus outbreaks reported in the eastern region was greater than in the western region. The high population density and mobility can accelerate the spread of rotavirus (11), and humidity and temperature in the southeastern coastal areas are also important factors affecting the outbreak of rotavirus (13). In addition, the rotavirus surveillance and laboratory tests were carried out earlier in the eastern provinces (15, 16).

This study showed that rotavirus outbreaks affected all age groups. Group B rotavirus outbreaks were mainly caused by contaminated water and affected populations of all age groups. On the other hand, Group A rotavirus outbreaks were primarily caused by person-to-person transmission and affected children. The positive rate of Group B rotavirus in sporadic surveillance was extremely low (17), and no outbreaks have been reported globally in recent years. Group A rotavirus is a major cause of acute gastroenteritis in infants and young children (18–20), and the outbreaks reported abroad primarily occurred in neonatal units (21) and in adults occasionally (22). The genotypes that cause rotavirus outbreaks may be common genotypes, such as G2P[4] (22), or rare genotypes, such as G12P[11] (23). Therefore, the genotypes of pathogens are crucial for analyzing infection characteristics and outbreak tracing.

Unlike norovirus, sapovirus, and astrovirus outbreaks, which occurred more frequently in nursery centers and kindergartens (24–27), rotavirus outbreaks mainly occurred in hospitals and villages. Vomiting is a common clinical symptom in children, and diarrhea is common in adults after norovirus infection (28, 29). Abdominal pain is the most common clinical symptom of astrovirus infection (24). However, diarrhea is the most prominent clinical symptom in children and adults infected with rotaviruses. Therefore, it can provide a reference for differential diagnosis.

The duration, number of cases, and attack rates of rotavirus outbreaks were significantly related to the types of settings. In this study, rotavirus outbreaks mainly occurred in villages and hospitals. Outbreaks in villages were already large when reported, caused mainly by waterborne transmission, had longer duration and greater numbers of cases, and had low attack rates due to the large number of people affected. In contrast, outbreaks in hospitals had a shorter duration, fewer cases, and higher attack rates. Nosocomial and community-acquired rotavirus outbreaks involved different settings and populations, thus influencing the attack rates and transmission modes. Due to the crowded nature of hospitals, they were mainly caused by person-to-person transmission with higher attack rates. However, due to the particularity of the hospital settings, early detection and timely professional management can shorten the duration of outbreaks and reduce the number of cases (22).

Waterborne outbreaks mainly occurred in villages before 1990, with more cases and longer duration, mostly caused by Group B rotaviruses. Due to economic development and improved water sanitation, waterborne rotavirus outbreaks are rarely reported. After 1990, rotavirus outbreaks mainly occurred in hospitals, mostly caused by Group A rotaviruses, and person-to-person transmission was most common. Hospitals have high population mobility, more sources of infection, and a large number of susceptible people, which is conducive to disease transmission. Therefore, continuous monitoring and intervention of nosocomial infection is essential.

Our study has several limitations. First, we only included rotavirus outbreaks reported in online literature, representing only a fraction of those that occurred in China. Second, the data for some indicators were not available for all outbreaks, for example, clinical symptoms of cases were not reported in every outbreak. Third, the age of some patients was missing, and the age groups in some studies were inconsistent, making detailed age distribution of the patients’ analysis impossible. Finally, most studies provided the groups of rotaviruses, but only a few studies provided rotavirus genotypes.

## 5 Conclusion and recommendations

This is the first systematic review based on the large number of rotavirus outbreaks in China reported in the literature. Historically, more waterborne rotavirus outbreaks have occurred in villages, with long durations and high numbers of cases. While rotavirus outbreaks have been rare in recent years, but they should still be taken into account in the investigation of acute gastroenteritis outbreaks investigation, especially for norovirus-negative outbreaks.

## Data availability statement

The original contributions presented in the study are included in the article/Supplementary material, further inquiries can be directed to the corresponding author.

## Author contributions

YT: Formal analysis, Investigation, Writing – original draft. FY: Formal analysis, Writing – original draft. GZ: Data curation, Investigation, Writing – review & editing. CT: Investigation, Writing – original draft. XW: Data curation, Investigation, Writing – review & editing. YC: Data curation, Investigation, Writing – review & editing. HY: Writing – review & editing. LJ: Writing – review & editing. DZ: Writing – review & editing. QW: Writing – review & editing. ZG: Formal analysis, Methodology, Writing – review & editing.

## Footnotes

^1^ https://www.rstudio.com/products/rstudio/download/

^2^ https://www.resdc.cn/data.aspx?DATAID=200
