# Supplementary material for: Rotavirus outbreaks in China, 1982–2021: a systematic review
Source: Front Public Health. 2024 Aug 8;12:1423573. doi: 10.3389/fpubh.2024.1423573 (PMC11338804; doi:10.3389/fpubh.2024.1423573)
Supplement: Supplementary file 1 [file Table_1.DOCX]

Supplementary Table 1.

Characteristics of included rotavirus outbreaks.

| **Number** | **Provinces** | **Onset time (year/month/day)** | **Settings** | **Duration** | **Exposed people** | **Cases** | **Attack rate (%)** | **Genogroups/**  **Genotypes** | **Transmission modes** |
| --- | --- | --- | --- | --- | --- | --- | --- | --- | --- |
| 1 | Heilongjiang | 1987/4/29 | Village | 35 | 483 | 157 | 32.51 | Group B | Water-borne |
| 2 | Guandong | 2002/1/4 | Army | 10 | 362 | 134 | 37.02 | Group B | Water-borne |
| 3 | Guandong | 2001/1/10 | Army | 11 | 41 | 25 | 60.98 | NA | Water-borne |
| 4 | Henan | 1996/10 | NA | 46 | 2050 | 384 | 18.73 | Group B | Water-borne |
| 5 | Guandong | 2004/6/27 | Hospital | 6 | 73 | 19 | 26.03 | Group A/G1 | Person-to-person |
| 6 | Guandong | 2004/7/28 | Hospital | 6 | 36 | 11 | 30.56 | Group A/G2 | Person-to-person |
| 7 | Liaoning | 1987 | NA | NA | NA | 156 | NA | NA | Water-borne |
| 8 | Guandong | 1994/4/7 | Hospital | 23 | 322 | 86 | 26.71 | NA | Person-to-person |
| 9 | Hunan | 1983/3/12 | Village | 76 | 4805 | 641 | 13.34 | Group B | Water-borne |
| 10 | Anhui | 1983/3 | Village | NA | NA | 20000 | NA | Group B | Water-borne |
| 11 | Shandong | 1986/5/7 | Army | 12 | 723 | 306 | 42.32 | Group B | Water-borne |
| 12 | Heilongjiang | 1988/2/16 | NA | 14 | 23108 | 3004 | 13.00 | Group B | Water-borne |
| 13 | Anhui | 1996/2/28 | Village | 17 | NA | 56 | NA | NA | Person-to-person |
| 14 | Shandong | 1983 | Factory and workers’ living place | NA | 871 | 149 | 17.11 | Group B | Person-to-person |
| 15 | Guangxi Zhuang Autonomous Region | 1993/8/18 | Factory and workers’ living place | 20 | 774 | 149 | 19.25 | Group B | Water-borne |
| 16 | Xinjiang Uygur Autonomous Region | 2019/5/7 | Army | 3 | 150 | 14 | 9.33 | Group A | Food-borne |
| 17 | Henan | 1990/1/25 | Village | 106 | 75428 | 2993 | 3.97 | Group B | Water-borne |
| 18 | Guangxi Zhuang Autonomous Region | 2010/10/15 | Hospital | 16 | NA | 8 | NA | Group A | Person-to-person |
| 19 | Guandong | 2006/10/1 | Village | 71 | 496 | 86 | 17.34 | NA | Multiple |
| 20 | Shanghai | 2006/7/22 | Hospital | 11 | NA | 26 | NA | Group B | Person-to-person |
| 21 | Hebei | 1997/4/10 | Schools | 19 | 9800 | 1055 | 10.77 | Group B | Water-borne |
| 22 | Jiangxi | 2017/3/14 | Schools | 18 | 12640 | 148 | 1.17 | Group A/G2P[4] | Food-borne |
| 23 | Shanxi | 1983/2 | Village | NA | NA | 6372 | NA | Group B | Water-borne |
| 24 | Shanxi | 1983/2 | Village | NA | NA |  | NA | Group B | Water-borne |
| 25 | Shanxi | 1983/6 | Village | NA | NA |  | NA | Group B | Water-borne |
| 26 | Shanxi | 1984/2 | Factory and workers’ living place | NA | NA |  | NA | Group B | Water-borne |
| 27 | Shanxi | 1984/2 | Factory and workers’ living place | NA | NA |  | NA | Group B | Water-borne |
| 28 | Shandong | 2013/10/21 | Hospital | 8 | NA | 5 | NA | NA | Person-to-person |
| 29 | Zhejiang | 2006/1/16 | Hospital | 4 | NA | 6 | NA | Group A/G1 | NA |
| 30 | Heibei | 2005/11/27 | Hospital | 6 | 112 | 29 | 25.89 | Group A/G1 | Person-to-person |
| 31 | Shandong | 1999/4/12 | Factory and workers’ living place | 35 | 1639 | 216 | 13.18 | NA | Water-borne |
| 32 | Jiangxi | 2013/4/16 | Hospital | 3 | NA | 3 | NA | NA | Person-to-person |
| 33 | Liaoning | 1982/12/1 | Factory and workers’ living place | 51 | 51531 | 7369 | 14.30 | Group B | Water-borne |
| 34 | Zhejiang | 2008/10/20 | Hospital | 7 | 106 | 46 | 43.40 | NA | NA |
| 35 | Henan | 1983/11/22 | Village | 19 | NA | 96 | NA | Group A | Multiple |
| 36 | Anhui | 2018/12/30 | Hospital | 6 | NA | 4 | NA | NA | Person-to-person |
| 37 | Beijing | 1997/11/19 | Hospital | 7 | 48 | 15 | 31.25 | Group A/G4P[2] | Person-to-person |
| 38 | Sichuan | 2012/7/7 | Hospital | 8 | 45 | 8 | 17.78 | NA | Person-to-person |
| 39 | Jiangsu | 2009/4/7 | Welfare house | 2 | 47 | 18 | 38.30 | NA | Person-to-person |
| 40 | Xinjiang Uygur Autonomous Region | 2008/9/25 | Hospital | 6 | NA | 12 | NA | NA | Person-to-person |
| 41 | Jiangxi | 2000/4/27 | Factory and workers’ living place | 17 | 1643 | 126 | 7.67 | NA | Water-borne |
| 42 | Liaoning | 2007/4/30 | Community | 17 | NA | 133 | NA | NA | Water-borne |
| 43 | Guangxi Zhuang Autonomous Region | 1983/7/19 | Factory and workers’ living place | 61 | 5959 | 657 | 11.03 | Group B | Water-borne |
| 44 | Shandong | 2003/4/19 | Factory and workers’ living place | 32 | 1853 | 468 | 25.26 | Group B | Water-borne |
| 45 | Inner Mongolia Autonomous Region | 2002/5/28 | Schools | 19 | NA | 610 | NA | Group B | Person-to-person |
| 46 | Guangdong | 1987/9/7 | Village | 34 | 704 | 79 | 11.22 | Group B | Water-borne |
| 47 | Jiangsu | 2012/2/21 | Village | 7 | 588 | 7 | 1.19 | Group A | Multiple |
| 48 | Zhejiang | 2012/12/18 | Schools | 9 | 1392 | 74 | 5.32 | Group A | Person-to-person |
| 49 | Hubei | 1986/5/4 | Village | 16 | 208 | 98 | 47.12 | Group B | NA |
| 50 | Hubei | 1988/11/1 | Village | 31 | 110 | 63 | 57.27 | Group A | Person-to-person |
| 51 | Yunnan | 1985/8/17 | Hospital | 104 | 372 | 190 | 51.08 | NA | Multiple |
| 52 | Yunnan | 2007/2/17 | Village | 18 | NA | 20 | NA | NA | Multiple |
| 53 | Hunan | 1983/3/16 | Village | 24 | 191 | 163 | 85.34 | Group B | Water-borne |
| 54 | Guangxi Zhuang Autonomous Region | 2006/11/26 | Community | 56 | 4006 | 137 | 3.42 | Group A/G1P[8] | Water-borne |
| 55 | Yunnan | 2004/12/15 | Village | 23 | 115 | 39 | 33.91 | Group A | NA |
| 56 | Yunnan | 2017/5/7 | Village | 19 | 435 | 44 | 10.11 | Group A | Water-borne |
| 57 | Hubei | 2004/2/13 | Schools | 9 | 2680 | 50 | 1.87 | NA | Food-borne |
| 58 | Hebei | 1989/3/1 | Village | 46 | 764 | 264 | 34.55 | Group B | Water-borne |
| 59 | Guangxi Zhuang Autonomous Region | 1985/10/23 | Schools | 39 | 1995 | 365 | 18.30 | Group B | Water-borne |
| 60 | Henan | 1985/4/20 | Village | 81 | 3050 | 296 | 9.70 | Group B | Water-borne |
| 61 | Guangxi Zhuang Autonomous Region | 1990/1/26 | Village | 16 | NA | 68 | NA | NA | Person-to-person |
| 62 | Jiangsu | 2019/2/22 | Schools | 13 | 921 | 26 | 2.82 | Group A | NA |
| 63 | Zhejiang | 2019/2/16 | Community | 12 | 11 | 5 | 45.45 | Group A/G9P[8] | Person-to-person |
| 64 | Shanxi | 2021/3/26 | Hospital | 11 | 59 | 6 | 10.17 | Group A | Person-to-person |
| 65 | Guangxi Zhuang Autonomous Region | 2020 | Village | NA | 686 | 26 | 3.79 | Group A | Water-borne |
| 66 | Beijing | 1994 | Schools | NA | NA | 400 | NA | NA | Water-borne |
| 67 | Liaoning | 1991/10/1 | Village | 43 | 19823 | 2232 | 11.26 | Group B | Water-borne |
| 68 | Zhejiang | 2015/2/19 | Village | 13 | 805 | 165 | 20.50 | NA | Water-borne |
| 69 | Jilin | 1987/5/25 | Army | 27 | 10125 | 970 | 9.58 | NA | Person-to-person |
| 70 | Hunan | 1983/4/24 | Factory and workers’ living place | 19 | 895 | 140 | 15.64 | NA | Water-borne |
| 71 | Shandong | 1985/11/7 | Hospital | 3 | NA | 8 | NA | Group A | Person-to-person |
| 72 | Heilongjiang | 1983 | Factory and workers’ living place | NA | 178 | 149 | 83.71 | Group A | Water-borne |
| 73 | Heilongjiang | 1984 | Restaurant | NA | 986 | 553 | 56.09 | Group A | Water-borne |
| 74 | Heilongjiang | 1985 | Schools | NA | 1024 | 289 | 28.22 | Group A | Water-borne |
| 75 | Liaoning | 1987/6/12 | Factory and workers’ living place | 18 | 56722 | 1367 | 2.41 | Group B | Water-borne |
| 76 | Shandong | 1984/3/8 | Army | 19 | 1733 | 643 | 37.10 | Group B | Water-borne |
| 77-82 | Guangxi Zhuang Autonomous Region | 1985/4 | Village | 62 | 2892 | 830 | 28.70 | Group B | Water-borne |
| 83 | Guandong | 2017/5/3 | Schools | 6 | 849 | 51 | 6.01 | Group A/G9P[8] | Person-to-person |
| 84 | Hebei | 1987/2/7 | Village | 27 | 518 | 165 | 31.85 | Group B | Person-to-person |
| 85 | Jilin | 1986/5/1 | NA | 61 | NA | 1000 | NA | Group B | Person-to-person |
| 86 | Guangxi Zhuang Autonomous Region | 1983/4/1 | NA | 154 | NA | 19007 | NA | NA | NA |
| 87 | Guangxi Zhuang Autonomous Region | 1984-6-1 | Village | NA | NA | 6570 | NA | NA | NA |
| 88 | Fujian | 1982/12/14 | Village | NA | 17000 | 261 | 1.54 | NA | NA |
| 89 | Yunnan | 1985/8/17 | Hospital | 104 | 372 | 190 | 51.08 | Group A | NA |
| 90 | Hong Kong，China | 1988/1/21 | Hospital | 12 | 125 | 7 | 5.60 | Group A | Person-to-person |
| 91 | Gansu | 1982/11/1 | Factory and workers’ living place | 93 | 46422 | 5942 | 12.80 | Group B | Person-to-person |
| 92 | Liaoning | 1982/12/5 | Factory and workers’ living place | 51 | 51712 | 7369 | 14.25 | Group B | Water-borne |
